# Non-linear relationship between red blood cell distribution width and gastrointestinal bleeding risk in stroke patients: results from multi-center ICUs

**DOI:** 10.3389/fneur.2024.1346408

**Published:** 2024-06-28

**Authors:** Zhanxing Wu, Ganggang Peng, Zhongqing Chen, Xiaoyong Xiao, Zhenhua Huang

**Affiliations:** Department of Emergency Medicine, The First Affiliated Hospital of Shenzhen University, Shenzhen Second People’s Hospital, Shenzhen, China

**Keywords:** non-linear relationship, red blood cell distribution width, gastrointestinal bleeding, stroke, multi-center retrospective study

## Abstract

**Background:**

The red blood cell distribution width (RDW) is closely linked to the prognosis of multiple diseases. However, the connection between RDW and gastrointestinal bleeding (GIB) in stroke patients is not well understood. This study aimed to clarify this association.

**Methods:**

This retrospective study involved 11,107 hospitalized patients from 208 hospitals in the United States, admitted between January 1, 2014, and December 31, 2015. We examined clinical data from 7,512 stroke patients in the intensive care unit (ICU). Multivariate logistic regression assessed the link between RDW and in-hospital GIB in stroke patients. Generalized additive model (GAM) and smooth curve fitting (penalty spline method) were utilized to explore the non-linear relationship between RDW and GIB in stroke patients. The inflection point was calculated using a recursive algorithm, and interactions between different variables were assessed through subgroup analyses.

**Results:**

Among the 11,107 screened stroke patients, 7,512 were included in the primary analysis, with 190 identified as having GIB. The participants had a mean age of (61.67 ± 12.42) years, and a median RDW of 13.9%. Multiple logistic analysis revealed RDW as a risk factor for in-hospital GIB in stroke patients (OR = 1.28, 95% CI 1.21, 1.36, *p* < 0.05). The relationship between RDW and in-hospital GIB in stroke patients was found to be non-linear. Additionally, the inflection point of RDW was 14.0%. When RDW was ≥14.0%, there was a positive association with the risk of GIB (OR: 1.24, 95% CI: 1.16, 1.33, *p* < 0.0001). Conversely, when RDW was <14.0%, this association was not significant (OR: 1.02, 95% CI: 0.97–1.07, *p* = 0.4040).

**Conclusion:**

This study showed a substantial non-linear link between RDW and the risk of GIB in stroke patients. Maintaining the patient’s RDW value below 14.0% could lower the risk of in-hospital GIB.

## Introduction

Gastrointestinal bleeding (GIB) is a frequent complication post-stroke, markedly increasing patient mortality (1–3). Studies show a 1.24% incidence of GIB after ischemic stroke in the United States and a 2.63% chance following hemorrhagic stroke in Japan (4, 5). In China, approximately 2.6% of hospitalized stroke patients experience GIB (5). Additionally, GIB has been associated with higher mortality risk in acute cardiovascular conditions such as Acute Coronary Syndrome (6, 7). Furthermore, GIB results in negative outcomes for stroke patients, with those suffering from ischemic stroke experiencing a 46% higher likelihood of severe disability and an 82% greater risk of in-hospital death (4). Our data indicate an 18.95% in-hospital mortality rate for stroke patients with GIB, a significantly high figure. Given the alarming link between GIB and stroke, identifying modifiable predictors of GIB, and enhancing patient outcomes are crucial.

Red blood cell distribution width (RDW) is a measurable clinical parameter routinely used to assess erythrocyte size variability and differentiate types of anemia. Over the past decade, RDW has gained attention as a predictive marker, confirmed by numerous studies. Research has shown a significant correlation between RDW and various diseases, including diabetes, chronic obstructive pulmonary disease (COPD), cardiovascular conditions, pneumonia, thromboembolism, Inflammatory Bowel Disease (IBD), and liver disorders (8–12), highlighting RDW’s prognostic value (13, 14).

Research indicates RDW is a valuable biomarker for assessing GIB status and prognosis (15). Liao et al. (16) in a retrospective study of 4,473 patients undergoing Coronary Artery Bypass Grafting (CABG) found elevated RDW levels associated with an increased risk of GIB post-CABG. Additionally, the study highlights the potential of RDW as a biomarker for assessing GIB risk following CABG surgery.

The role of RDW as an independent predictor of GIB in stroke patients remains to be further investigated. This study aims to investigate the relationship between RDW and in-hospital GIB following a stroke, and assess RDW’s predictive utility for this condition.

## Methods

### Data source

This study utilized data from the eICU Collaborative Research Database (eICU-CRD), a multi-center intensive care database comprising over 200,000 cases (17, 18). Electronic medical records from 208 U.S. hospitals, spanning 2014 to 2015, were compiled. The Institutional Review Board of the Massachusetts Institute of Technology (Cambridge, Massachusetts, USA) granted approval for database use. Data access and extraction were conducted by the author, Zhenhua Huang, under certification number 499395491.

### Study population

**Figure 1:**
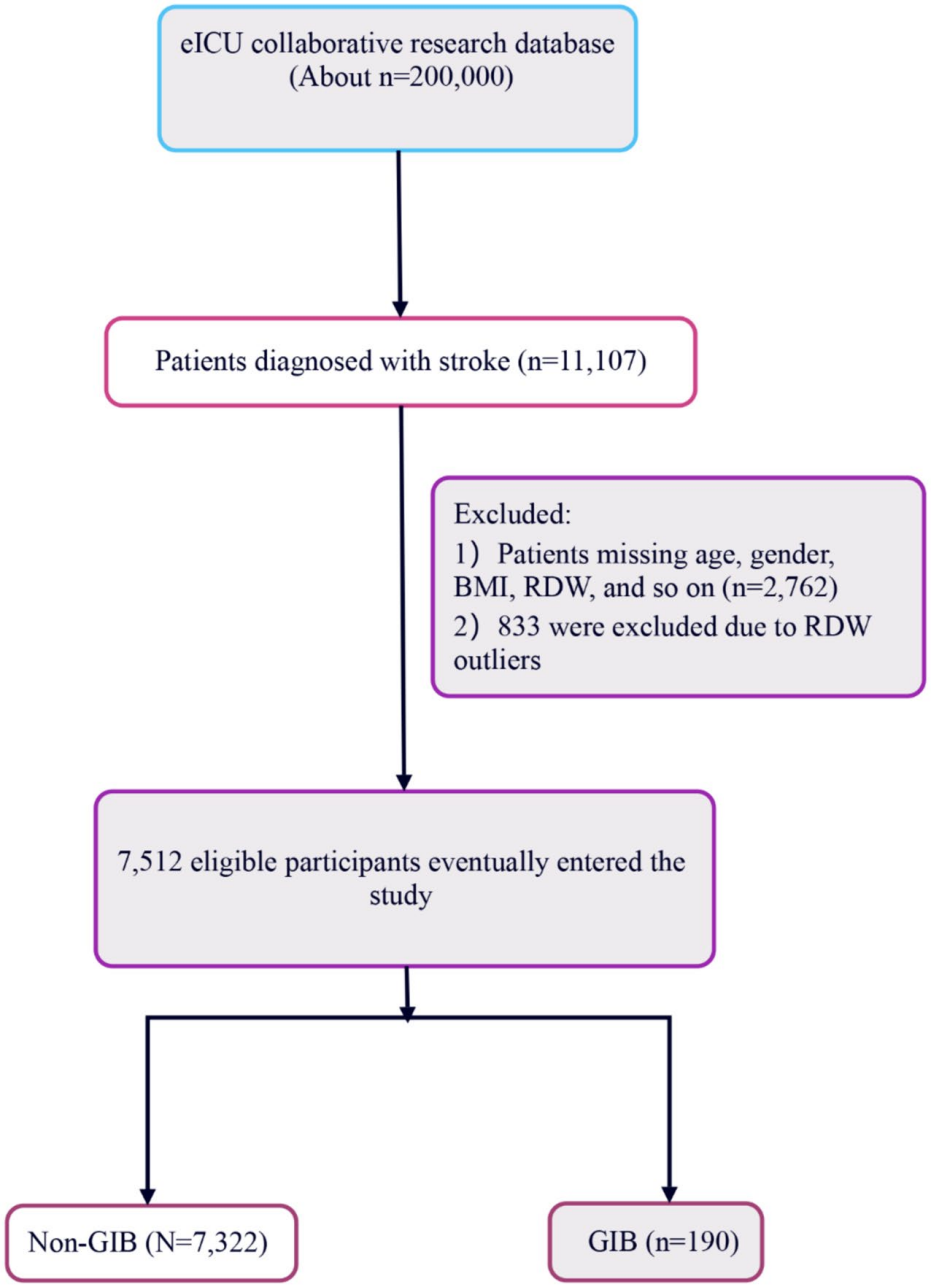
Flowchart of study participants.

Patients diagnosed with stroke, as recorded in the patient dataset, were potentially eligible. These stroke patients were classified into three groups: the ischemic stroke (IS) group, the hemorrhagic stroke (HS) group (comprising patients with subarachnoid hemorrhage and intracerebral hemorrhage), and another group. Initially, 11,107 stroke patients were included in this study. Subsequently, 2,762 patients were excluded due to missing data on factors such as age, gender, BMI, RDW. 833 were excluded due to RDW outliers. And those who experienced GIB within 24 h of admission or prior to admission were also excluded. Ultimately, the cohort comprised 7,512 stroke patients, including 2,371HS patients, 2,608 IS patients, and 2,533 patients with other stroke types. Among these, 190 patients were identified with GIB during their hospital stay (as depicted in Figure 1). When multiple measurements of RDW and other variables were taken after admission to the ICU, data from the initial measurement were utilized.

### Variable extraction

In this study, the main outcome measured was the occurrence of in-hospital GIB of hospitalization for stroke patients. Detailed demographic data, such as age, body mass index (BMI), red blood cell count (RBC), hemoglobin (Hb), platelet count (PC), RDW, blood urea nitrogen (BUN), blood calcium, serum creatinine (Scr), gender, ethnicity, and past medical history, were gathered. Furthermore, for variables that were recorded multiple times within the first 24 h of admission, the value most strongly linked to disease severity was chosen (as shown in Table 1).

**Table 1:** Baseline characteristics of participants.

RDW (%) (quartile)	Q1 (11.4–13.1)	Q2 (13.2–13.8)	Q3 (13.9–14.9)	Q4 (15.0–24.8)	*p*-value
*N*	1,818	1,859	1,850	1,985	
Age (year)	58.35 ± 13.17	61.93 ± 12.19	63.37 ± 11.56	62.89 ± 12.13	<0.001
BMI (kg/m^2^)	27.73 ± 7.78	28.39 ± 8.49	29.02 ± 9.00	29.02 ± 9.98	<0.001
RBC (× 10^12^/L)	4.48 ± 0.59	4.45 ± 0.66	4.34 ± 0.76	4.02 ± 0.93	<0.001
Hb (g/L)	139.1 ± 17.8	135.8 ± 19.6	129.5 ± 22.2	112.8 ± 24.8	<0.001
PC (× 10^9^/L)	219.0 (182.0–260.0)	218.0 (174.5–263.0)	217.0 (174.0–270.0)	223.0 (167.0–288.0)	<0.001
BUN (mmol/L)	14.0 (11.0–19.0)	15.0 (12.0–21.0)	17.0 (13.0–24.0)	20.0 (14.0–34.0)	<0.001
Calcium (mg/dL)	8.52 ± 1.98	8.61 ± 1.73	8.55 ± 1.81	8.47 ± 1.70	0.110
Scr (mg/dL)	0.84 (0.69–1.06)	0.90 (0.70–1.10)	0.97 (0.74–1.31)	1.09 (0.80–1.70)	<0.001
Gender, *n* (%)					<0.001
Male	1,049 (57.70%)	1,039 (55.89%)	1,026 (55.46%)	1,005 (50.63%)	
Female	769 (42.30%)	820 (44.11%)	824 (44.54%)	980 (49.37%)	
Ethnicity, *n* (%)					<0.001
African American	140 (7.70%)	176 (9.47%)	306 (16.54%)	442 (22.27%)	
Asian	57 (3.14%)	43 (2.31%)	33 (1.78%)	28 (1.41%)	
Caucasian	1,420 (78.11%)	1,397 (75.15%)	1,325 (71.62%)	1,337 (67.36%)	
Hispanic	75 (4.13%)	84 (4.52%)	63 (3.41%)	77 (3.88%)	
Native American	3 (0.17%)	9 (0.48%)	15 (0.81%)	15 (0.76%)	
Unknown	123 (6.77%)	150 (8.07%)	108 (5.84%)	86 (4.33%)	
History, *n* (%)					
AF	120 (6.60%)	137 (7.37%)	190 (10.27%)	280 (14.11%)	<0.001
ACS	44 (2.42%)	83 (4.46%)	93 (5.03%)	117 (5.89%)	<0.001
CHF	20 (1.10%)	56 (3.01%)	83 (4.49%)	174 (8.77%)	<0.001
COPD	180 (9.90%)	199 (10.70%)	303 (16.38%)	355 (17.88%)	<0.001
Diabetes	193 (9.97%)	196 (9.53%)	356 (16.01%)	371 (17.43%)	<0.001
Hypertension	531 (27.43%)	656 (31.89%)	752 (33.81%)	652 (30.64%)	<0.001
Cancer	516 (28.38%)	597 (32.11%)	635 (34.32%)	607 (30.58%)	0.01
Stroke type, *n* (%)					0.964
Hemorrhagic stroke	563 (30.97%)	587 (31.58%)	601 (32.49%)	620 (31.23%)	
Ischemic stroke	642 (35.31%)	638 (34.32%)	637 (34.43%)	691 (34.81%)	
Others	613 (33.72%)	634 (34.10%)	612 (33.08%)	674 (33.95%)	
In-hospital GIB, *n* (%)	21 (1.16%)	20 (1.08%)	41 (2.22%)	108 (5.44%)	<0.001
Status at hospital discharge, *n* (%)					<0.001
Alive	1,666 (91.64%)	1,648 (88.65%)	1,620 (87.57%)	1,601 (80.65%)	
Death	152 (8.36%)	211 (11.35%)	230 (12.43%)	384 (19.35%)	

Continuous variables are summarized as mean (SD) or median (quartile interval); categorical variables are presented as percentages (%). BMI, body mass index; BUN, blood urea nitrogen; Scr, serum creatinine; RBC, red blood cell; Hb, hemoglobin; PC, platelet count; ACS, acute coronary syndrome; AF, atrial fibrillation; CHF, congestive heart-failure; COPD, chronic obstructive pulmonary disease; GIB, gastrointestinal bleeding.

### Statistical analysis

RDW was categorized into four quartiles: Q1 (11.4–13.1%), Q2 (13.2–13.8%), Q3 (13.9–14.9%), and Q4 (15.0–24.8%). Descriptive statistics were provided for continuous variables, including the mean and standard deviation for normally distributed data, medians with interquartile range (IQR) for non-normally distributed data, and for categorical variables, frequency and proportion were reported. Various statistical tests were employed to compare different RDW groups based on the distribution of the data. One-way analysis of variance was used for groups with a normal distribution, the χ2 method for categorical variables, and the Kruskal-Wallis H test for groups with a non-normal distribution. These tests were selected appropriately to accurately analyze the data and draw valid conclusions based on the diverse characteristics of the RDW groups. Logistic regression analysis was performed to explore the association between RDW and hospital-acquired GIB in stroke patients. Adjusted odds ratios (OR) and 95% confidence intervals (CI) were calculated for the study results using multivariable models. Three models were constructed: (i) an unadjusted model, (ii) a minimally adjusted model (Model I: adjusted for age, gender, and ethnicity), and (iii) a fully adjusted model (adjusted for gender, age, ethnicity, atrial fibrillation (AF), congestive heart failure (CHF), acute coronary syndrome (ACS), COPD, diabetes, hypertension, and cancer). Given the suspicion that binary logistic regression models might not adequately handle nonlinear relationships, we explored such relationships using generalized additive models (GAM) and smooth curve fitting (penalized spline method). If nonlinearity was detected, we initially calculated the inflection point using a recursive algorithm and then established a two-piecewise linear logistic regression model on both sides of the inflection point. The log-likelihood ratio test was employed to determine the most suitable model describing the association between RDW and GIB. Additionally, interactions between different variables were assessed through subgroup analyses. ROC curve analysis was conducted to assess the predictive capability of RDW for in-hospital GIB among stroke patients, with a significance threshold set at *p* < 0.05. The analysis was conducted using Empower Stats software and the R language, with statistical significance defined as a two-tailed *p*-value <0.05.

## Results

### Baseline characteristics

**Figure 2:**
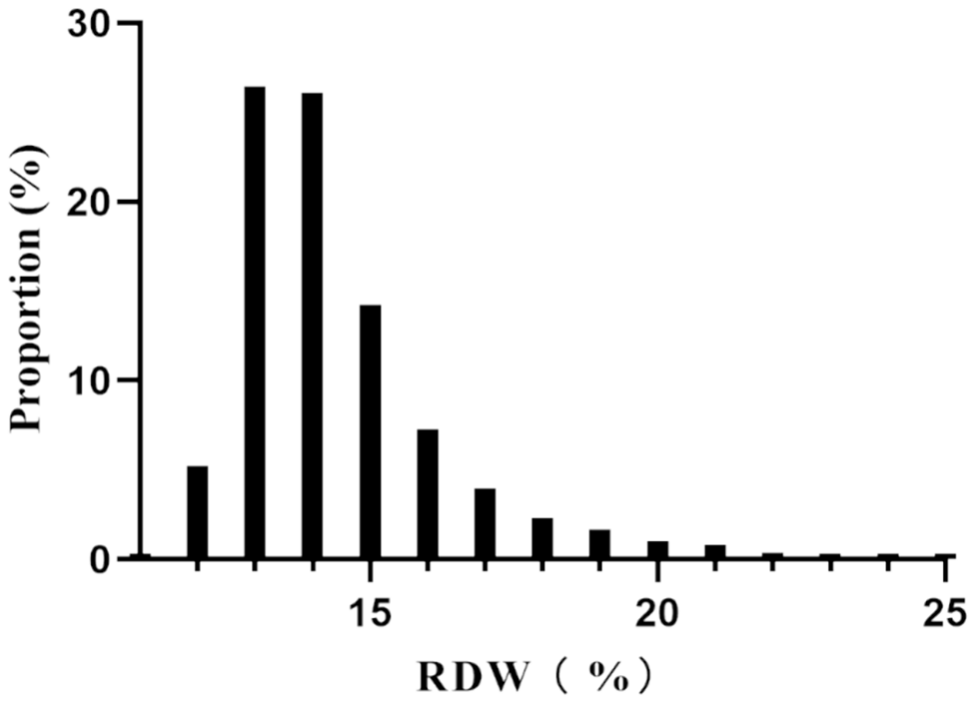
Distribution of RDW. It showed that RDW presented a skewed distribution ranging from 11.4 to 24.8%, with a median level of 13.9%.

The participants were divided into quartiles, each defined by a range of RDW values. When comparing these quartiles to the Q1 reference group, several variables showed significant differences. In the highest quartile (Q4: RDW ≥ 15%), age, BMI, PC, BUN, and Scr were notably higher. Additionally, elevated rates of AF, CHF, ACS, COPD, and hypertension were observed in this quartile. Conversely, the proportion of males, Hb, calcium and RBC were lower in the highest quartile (all *p*-values <0.05). Furthermore, both hospital mortality and the incidence of GIB significantly increased in Q4 compared to Q1 (p-values <0.05) (Table 1). Figure 2 illustrates the skewed distribution of RDW, ranging from 11.4 to 24.8%, with a median level of 13.9%.

### Factors influencing in-hospital GIB risk in stroke patients via univariate analysis

Table 2 illustrates the correlations between various factors and GIB following hospitalization for stroke patients, as analyzed through univariate analysis. Significant associations with GIB (*p* < 0.05) were observed for RDW, age, Scr, calcium, BUN, RBC, Hb and BUN levels. Similarly, GIB exhibited significant correlations with ACS, COPD, CHF, AF, diabetes, and hypertension rates (all p < 0.05). However, no significant associations were found between hospital-acquired GIB and BMI, PC, gender, ethnicity, or cancer rates (all *p* > 0.05).

**Table 2:** Factors influencing risk of in-hospital GIB in stroke patients analyzed by univariate analysis.

	Statistics	OR (95% CI)	*p*-value
Gender, *n* (%)			0.1478
Male	4,119 (54.83%)	1.0	
Female	3,393 (45.17%)	0.80 (0.60, 1.08)	
Age (years)	61.67 ± 12.42	1.02 (1.00, 1.03)	0.0067
Ethnicity, *n* (%)			
African American	1,064 (14.16%)	1.0	
Asian	161 (2.14%)	1.17 (0.48, 2.84)	0.7240
Caucasian	5,479 (72.94%)	0.71 (0.49, 1.05)	0.0848
Hispanic	299 (3.98%)	1.05 (0.51, 2.15)	0.8975
Native American	42 (0.56%)	0.00 (0.00, Inf)	0.9717
Unknown	467 (6.22%)	0.94 (0.50, 1.76)	0.8381
BMI, (kg/m^2^)	28.55 ± 8.89	1.00 (0.99, 1.02)	0.6382
AF, *n* (%)	727 (9.68%)	2.24 (1.55, 3.25)	<0.0001
ACS, *n* (%)	337 (4.49%)	3.40 (2.20, 5.26)	<0.0001
CHF, *n* (%)	333 (4.43%)	2.95 (1.87, 4.67)	<0.0001
COPD, *n* (%)	321 (4.27%)	3.25 (2.07, 5.10)	<0.0001
Diabetes, *n* (%)	1,037 (13.80%)	2.23 (1.60, 3.10)	<0.0001
Hypertension, *n* (%)	2,355 (31.35%)	2.50 (1.87, 3.33)	<0.0001
Cancer, *n* (%)	68 (0.91%)	1.79 (0.56, 5.75)	0.3274
PC, (× 10^9^/L)	219.0 (175.0–270.0)	1.00 (1.00, 1.00)	0.0505
RBC, (× 10^12^/L)	4.32 ± 0.77	0.40 (0.34, 0.48)	<0.0001
Hb, (g/L)	129.0 ± 23.7	0.72 (0.68, 0.76)	<0.0001
RDW, (%)	14.39 ± 1.88	1.28 (1.22, 1.35)	<0.0001
BUN, (mmol/L)	16.0 (12.0–24.0)	1.03 (1.02, 1.03)	<0.0001
Calcium, (mg/dL)	8.54 ± 1.81	0.93 (0.87, 0.99)	0.0196
Scr, (mg/dL)	0.92 (0.72–1.24)	1.14 (1.07, 1.21)	<0.0001

OR and 95% CI were calculated for the study results using univariate models. OR, odds ratios; CI, confidence intervals.

### RDW’s association with in-hospital GIB in univariate and multivariate analyses

In univariate analysis, a significant association was observed between continuous RDW and in-hospital GIB (OR 1.28, 95% CI (1.22, 1.35), *p* < 0.001). To address various risk factors, including critical clinical variables, we developed a multivariate model. RDW remained an independent predictor of in-hospital GIB in both adjusted Model II (OR 1.29, 95% CI 1.22–1.36, *p* < 0.001) and Model III (OR 1.28, 95% CI 1.21–1.36, p < 0.001), as shown in Table 3. Furthermore, all participants were categorized into four groups based on RDW quartiles. Using the Q1 group as a reference, a significant increase in GIB risk was observed in the highest quartile (Q4) (*p* < 0.001), as illustrated in Table 3.

**Table 3:** Relationship between RDW and in-hospital GIB in stroke patients in different models.

Exposure	Model I (OR, 95%CI) *P*	Model II (OR, 95%CI) *P*	Model III (OR, 95%CI) *P*
RWD (%)	1.28 (1.22, 1.35) <0.0001	1.29 (1.22, 1.36) <0.0001	1.28 (1.21, 1.36) <0.0001
(quartile)			
Q1	Ref	Ref	Ref
Q2	0.93 (0.50, 1.72) 0.8190	0.90 (0.48, 1.66) 0.7294	0.83 (0.45, 1.54) 0.5546
Q3	1.94 (1.14, 3.29) 0.0143	1.84 (1.08, 3.14) 0.0250	1.65 (0.96, 2.83) 0.0687
Q4	4.92 (3.07, 7.89) <0.0001	4.76 (2.96, 7.65) <0.0001	4.23 (2.59, 6.90) <0.0001
P for trend	<0.0001	<0.0001	<0.0001

Adjusted model I: no covariates were adjusted for. Adjusted model II: we only adjusted for age, gender, and ethnicity. Adjusted model III: we adjusted for age, gender, ethnicity, AF, CHF, ACS, COPD, diabetes, hypertension, and cancer.

### Non-linear relationship of RDW and in-hospital GIB in stroke patients

**Figure 3:**
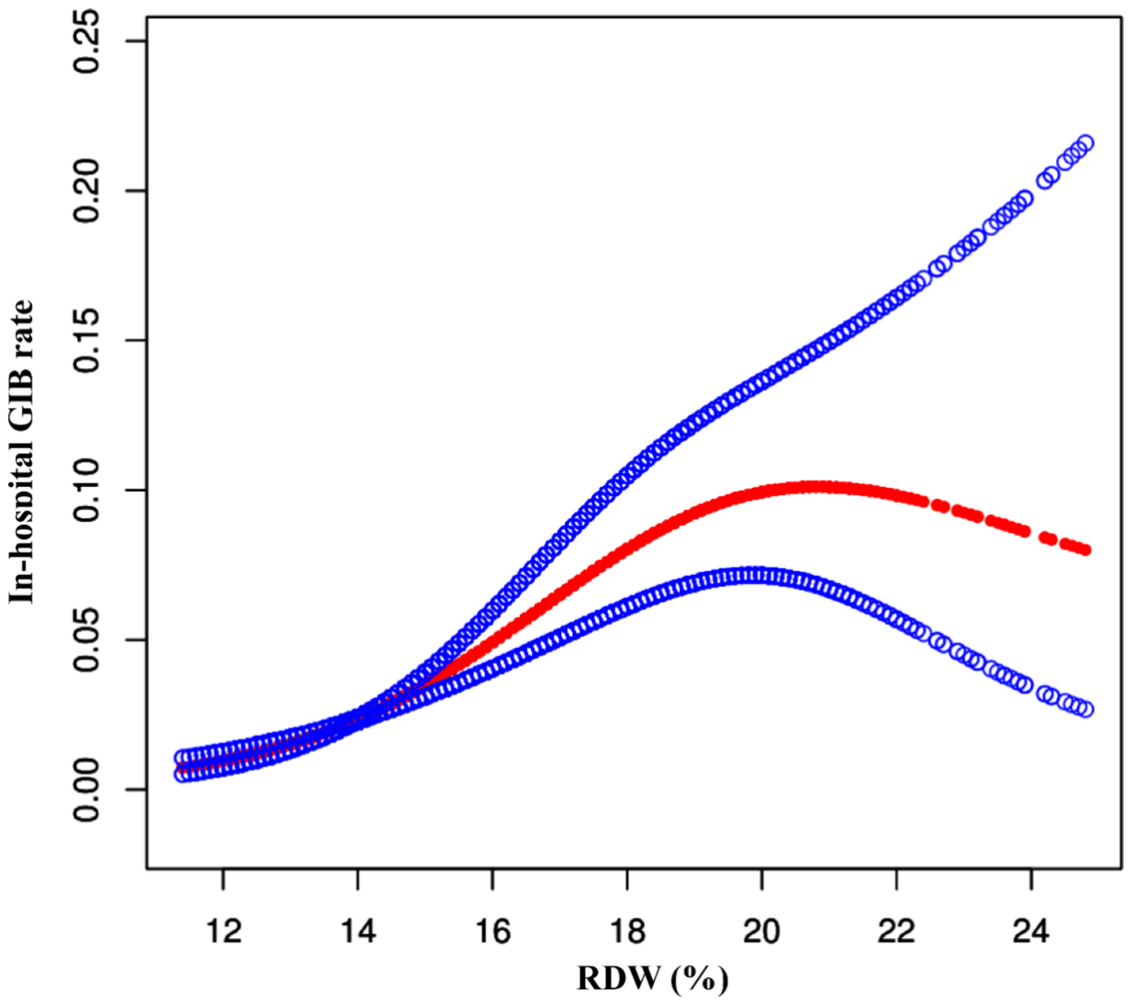
The nonlinear relationship between RDW and risk of GIB in stroke patients. A nonlinear relationship between them was detected after adjusting for age, gender, ethnicity, AF, CHF, ACS, COPD, diabetes, hypertension, and cancer.

To further explore the relationship between RDW and in-hospital GIB incidence, we generated smoothing curves using a generalized additive model, adjusting for age, gender, ethnicity, AF, CHF, ACS, COPD, diabetes, hypertension, and cancer. The results showed a nonlinear association between RDW and in-hospital GIB risk, as illustrated in Figure 3. Furthermore, the curve notably steepened in the Q4 quartile, indicating a rapid and significant increase in in-hospital GIB incidence with rising RDW, especially in the Q4 group. Segmented regression models identified a threshold RDW value of 14.0% (*p* < 0.001 based on the log-likelihood ratio test). RDW ≥ 14.0% was positively associated with GIB risk (OR: 1.24, 95% CI: 1.16, 1.33, *p* < 0.0001). Conversely, RDW < 14.0% showed no significant association with GIB risk (OR: 1.02, 95% CI: 0.97–1.07, *p* = 0.4040) (as demonstrated in Table 4).

**Table 4:** The result of two-piecewise linear regression model.

Outcome: GIB	OR, 95%CI	*p*-value
Fitting model by standard linear regression	1.28 (1.22, 1.35)	<0.0001
Fitting model by two-piecewise linear regression model		
Inflection points of RDW	14.0%	
<14.0%	1.02 (0.97, 1.07)	0.4040
≥14.0%	1.24 (1.16, 1.33)	<0.0001
*p* for log-likelihood ratio test		<0.001

We adjusted for age, gender, ethnicity, AF, CHF, ACS, COPD, diabetes, hypertension, and cancer.

### Subgroup analyses for RDW impact on in-hospital GIB after stroke

Subgroup analyses were conducted to identify potential factors influencing RDW’s impact on in-hospital GIB post-stroke, as outlined in Table 5. Significant interactions between RDW and BUN, as well as creatinine levels, were observed regarding in-hospital GIB post-stroke. Specifically, patients with BUN levels <20 mg/dL had an elevated risk of GIB (OR = 1.37, 95% CI 1.24, 1.51), while those with creatinine levels <1.2 mg/dL also showed increased GIB risk (OR = 1.38, 95% CI 1.26, 1.50). There were no significant interactions observed between age, RBC, AF, ACS, CHF, COPD, DM, DKA, cancer, stroke type, and in-hospital GIB.

**Table 5:** Stratified analyses of the association between RDW and in-hospital GIB.

Exposure	OR (95% CI)	*p*-value	*p* for interaction
Age (year)			0.9841
<60	1.28 (1.16, 1.41)	<0.0001	
≥60	1.28 (1.19, 1.38)	<0.0001	
Scr (mg/dL)			0.0102
<0.6	1.35 (1.11, 1.64)	0.0027	
≥0.6, <1.2	1.38 (1.26, 1.50)	<0.0001	
≥1.2	1.15 (1.06, 1.25)	0.0010	
BUN (mmol/L)			0.0546
<9	1.37 (1.09, 1.72)	0.0068	
≥9, <20	1.37 (1.24, 1.51)	<0.0001	
≥20	1.18 (1.10, 1.28)	<0.0001	
RBC (× 10^12^/L)			0.0820
<4	1.17 (1.09, 1.27)	<0.0001	
≥4	1.31 (1.19, 1.43)	<0.0001	
AF			0.4480
No	1.29 (1.21, 1.38)	<0.0001	
Yes	1.22 (1.07, 1.40)	0.0032	
ACS			0.6737
No	1.29 (1.21, 1.37)	<0.0001	
Yes	1.23 (1.03, 1.48)	0.0250	
CHF			0.6828
No	1.29 (1.21, 1.37)	<0.0001	
Yes	1.24 (1.05, 1.47)	0.0130	
COPD			0.2057
No	1.29 (1.22, 1.37)	<0.0001	
Yes	1.12 (0.89, 1.40)	0.3277	
DM			0.0998
No	1.31 (1.23, 1.40)	<0.0001	
Yes	1.17 (1.03, 1.33)	0.0142	
Cancer			0.2851
No	1.29 (1.21, 1.36)	<0.0001	
Yes	1.01 (0.62, 1.65)	0.9680	
Type of stroke			0.4631
Hemorrhagic stroke	1.33 (1.22, 1.46)	0.0002	
Ischemic stroke	1.22 (1.11, 1.36)	0.0220	
Other	1.28 (1.16, 1.42)	0.0005	

Above model adjusted for age, gender, ethnicity, AF, CHF, ACS, COPD, diabetes, hypertension, and cancer. In each case, the model is not adjusted for the stratification variable when the stratification variable was a categorical variable.

### Roc curves for RDW predicting in-hospital GIB after stroke

**Figure 4:**
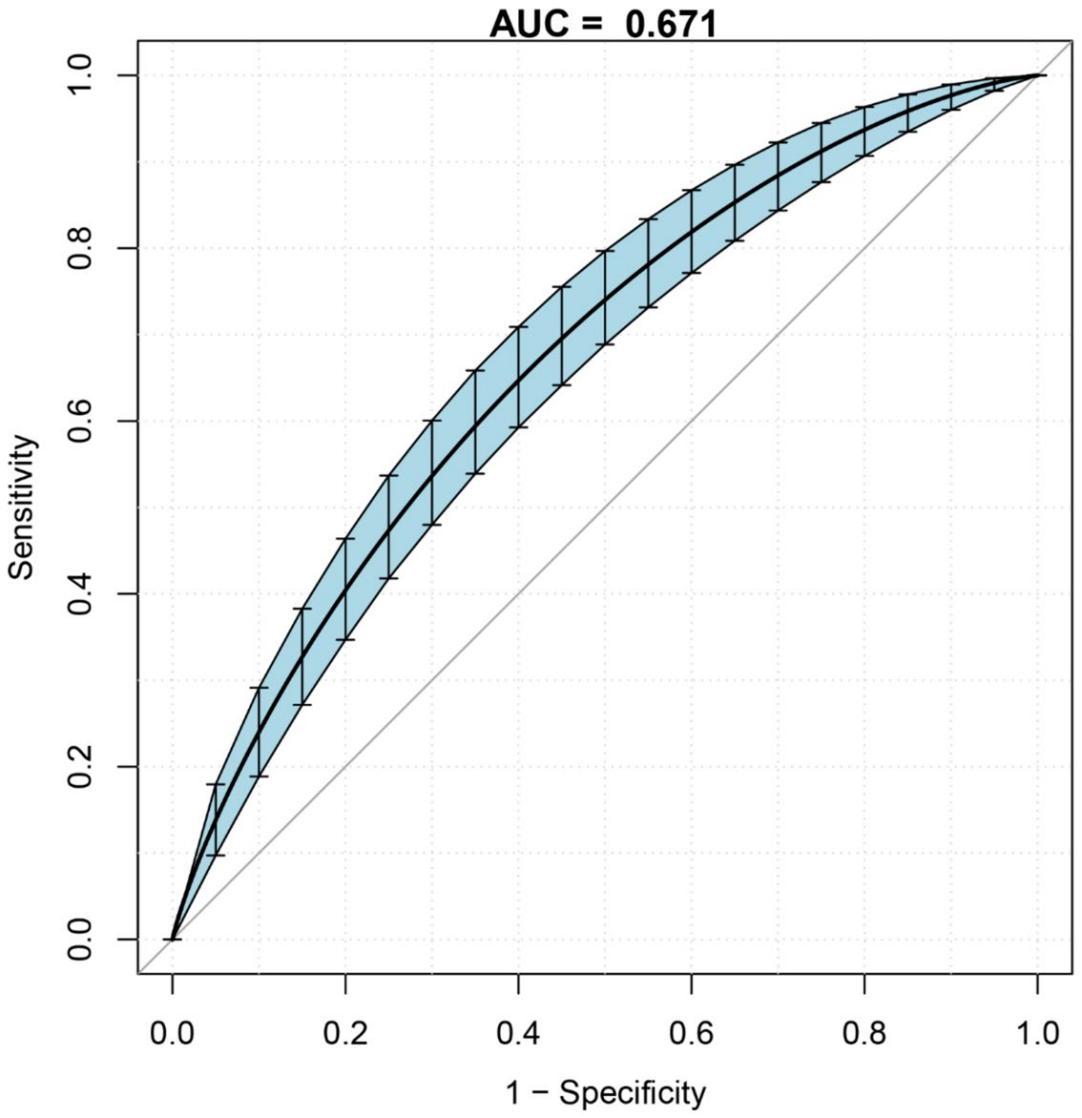
ROC curves for RDW in predicting in-hospital GIB in stroke patients.

Figure 4 and Table 6 presents the Receiver operating characteristics (ROC) curves for RDW in predicting in-hospital GIB in stroke patients among all participants. The AUC values and 95% CIs for RDW was 0.671 (0.632, 0.708). And the best threshold was 14.850.

**Table 6:** AUC with the 95% CI of RDW for predicting in-hospital GIB in stroke patients.

	AUC	95% CI low	95% CI up	Best threshold	Sensitivity (%)	Specificity (%)
RDW	0.671	0.632	0.708	14.850	72.8	59.5

## Discussion

Our retrospective study indicates a positive correlation between elevated RDW levels and increased risk of GIB in stroke patients. Specifically, each unit increase in RDW was associated with a 28% higher risk of GIB. We also discovered a non-linear correlation between RDW levels and GIB risk, where levels ≥14.0% showed a significant positive correlation (OR: 1.24, 95% CI: 1.16–1.33, *p* < 0.0001), while levels <14.0% did not show a statistically significant correlation (OR: 1.02, 95% CI:0.97–1.07, *p* = 0.4040).

Recent studies consistently demonstrate a strong link between increased RDW and a range of diseases such as cancers, digestive disorders, and cardiovascular conditions (19–23). Tonelli et al. (24) found that higher RDW levels correlate with increased cardiovascular events in patients with coronary artery disease, indicating that RDW may reflect disease severity and prognosis. Felker et al. (25) noted that higher RDW levels are associated with adverse outcomes in heart failure patients, underscoring its utility as a prognostic marker. Patel et al. (26) linked higher RDW levels to increased mortality in older adults, highlighting RDW’s role as a survival predictor, useful for health and prognosis evaluations. Recent studies have explored the link between higher RDW levels and increased GIB risk. A 2017 study showed that elevated RDW levels correlate with greater upper gastrointestinal bleeding (UGIB) risk (27). Another study identified RDW as an independent predictor of GIB post-CABG (16).

In 2019, stroke was a leading global health issue, with approximately 12.2 million cases worldwide, making it the second leading cause of death (28). GIB is a serious complication following a stroke, often presenting through subtle symptoms that pose challenges in timely diagnosis. Our study investigated the non-linear relationship between RDW and in-hospital GIB in stroke patients, even when adjusting for factors like age, gender, ethnicity, and various health conditions. Our analysis revealed significant interactions between RDW and both BUN and creatinine levels in relation to in-hospital GIB. Patients with lower BUN and creatinine levels demonstrated an increased risk of GIB, suggesting that monitoring RDW values can prompt early intervention and potentially reduce GIB incidence in stroke patients.

Our study identifies a non-linear association between RDW and in-hospital GIB in stroke patients, accounting for variables such as age, gender, ethnicity, and health conditions. Using a two-piecewise linear logistic regression model, we established the inflection point for RDW. At RDW levels ≥14.0%, there was a significant positive correlation with GIB risk. Conversely, at RDW levels <14.0%, the association was not significant. Subgroup analysis explored factors influencing RDW’s impact on in-hospital GIB in stroke patients. Significant interactions between RDW and both BUN and Scr levels were noted, relating to in-hospital GIB in stroke patients. Patients with BUN below 20 mg/dL and creatinine below 1.2 mg/dL showed an increased GIB risk. Therefore, using RDW values to predict GIB risk can facilitate early intervention by healthcare providers, potentially reducing GIB incidence in stroke patients.

Our study has notable strengths. For the first time, we explored the association between RDW and GIB risk in stroke patients, utilizing data from multiple centers and a large sample size. Through comprehensive multivariable logistic regression analysis, we accounted for several variables associated with GIB risk, including age, gender, ethnicity, and medical conditions such as AF, CHF, ACS, COPD, diabetes, hypertension, and cancer. This thorough consideration of pertinent variables bolsters the credibility of our findings. Secondly, we utilized cubic spline functions and smooth curve fitting to elucidate the non-linear relationship between RDW and GIB in stroke patients. Importantly, we identified the inflection point for RDW, offering critical clinical insights for preventing GIB in stroke patients.

However, our study has certain limitations. Firstly, this analysis relies on retrospective data, which may have limitations related to data collection and potential biases. We plan to conduct prospective research in the future to address these limitations. Secondly, the presence of only short-term follow-up data in the database limited our ability to discern long-term outcomes. Additionally, this retrospective observational study provides an association inference rather than establishing a causal relationship between RDW and The risk of gastrointestinal bleeding in stroke patients. Finally, given that our study is retrospective, the original database does not include data on non-ICU stroke patients. Therefore, we could not incorporate non-ICU stroke patients in this study. These limitations should be considered when interpreting our findings.

## Conclusion

Our study revealed a significant non-linear correlation between RDW and GIB risk in stroke patients. Our findings also highlight that keeping the patient’s RDW value below 14.0% may reduce the risk of in-hospital GIB in stroke patients.

## Data availability statement

The data analyzed in this study was obtained from the electronic Intensive Care Unit Collaborative Research Database (eICU-CRD), the following licenses/restrictions apply: to access the files, users must be credentialed users, complete the required training (CITI Data or Specimens Only Research) and sign the data use agreement for the project. Requests to access these datasets should be directed to PhysioNet, https://physionet.org/, DOI: 10.13026/0pzc-dm64.

## Ethics statement

Ethical review and approval was not required for the study on human participants in accordance with the local legislation and institutional requirements. Written informed consent from the patients/participants or patients/participants' legal guardian/next of kin was not required to participate in this study in accordance with the national legislation and the institutional requirements.

## Author contributions

ZW: Data curation, Writing – original draft. GP: Data curation, Formal analysis, Writing – original draft. ZC: Data curation, Writing – original draft. XX: Writing – review & editing. ZH: Data curation, Writing – review & editing.
